# Anxiety and Alcohol Use Disorders: Comorbidity and Treatment Considerations

**DOI:** 10.35946/arcr.v34.4.06

**Published:** 2012

**Authors:** Joshua P. Smith, Carrie L. Randall

**Affiliations:** **Joshua P. Smith, Ph.D.,***is an assistant professor at the Charleston Alcohol Research Center and outpatient director at the Center for Drug and Alcohol Programs, Department of Psychiatry and Behavioral Sciences, Medical University of South Carolina, Charleston, South Carolina.*; **Carrie L. Randall, Ph.D.,***is a Distinguished University Professor at the Charleston Alcohol Research Center, Center for Drug and Alcohol Programs, Medical University of South Carolina, Charleston, South Carolina.*

**Keywords:** Alcohol use disorders, stress, anxiety disorders, comorbidity, developmental pathway, treatment, treatment method, sequential approach, parallel approach, integrated approach

## Abstract

The co-occurrence of anxiety disorders and alcohol use disorders (AUDs) is relatively common and is associated with a complex clinical presentation. Sound diagnosis and treatment planning requires that clinicians have an integrated understanding of the developmental pathways and course of this comorbidity. Moreover, standard interventions for anxiety disorders or AUDs may need to be modified and combined in targeted ways to accommodate the unique needs of people who have both disorders. Optimal combination of evidence-based treatments should be based on a comparative balance that considers the advantages and disadvantages of sequential, parallel, and integrated approaches.

Co-occurring anxiety disorders and alcohol use disorders (AUDs) are of great interest to researchers and clinicians. Cumulative evidence from epidemiological and clinical studies over the past few decades has highlighted both the frequency and clinical impact of this comorbidity. Investigations into the unique connections between specific anxiety disorders and AUDs have shown that this association is multifaceted and complex, underscoring the importance of careful diagnostic scrutiny. Of clinical relevance, treatment for people with comorbid anxiety and AUDs can be complicated, and both the methods used and the timing of the interventions are relevant factors in treatment planning and delivery. This article explores the relationship between anxiety disorders and AUDs, focusing on the prevalence, clinical impact, developmental and maintenance characteristics, and treatment considerations associated with this fairly common comorbidity. The distinctive nature of the relationship between posttraumatic stress disorder (PTSD) and AUDs is discussed separately, in the article by Brady and Back, p. 408 in this journal issue.

## Prevalence and Clinical Impact of Comorbid Anxiety and AUDs

Accuracy in prevalence estimates of comorbid anxiety and AUDs is essential for gauging the magnitude of the clinical and social impact of this comorbidity; therefore, data should be carefully selected with attention to sampling methods. Information derived from clinical samples, although enlightening in its own right, produces inflated approximations of the prevalence of comorbidity (Kushner et al. 2008; Regier et al. 1990; Ross 1995). The most frequently offered explanation for the biased estimates from clinic-based samples suggests that individuals with multiple disorders are more likely to be referred for treatment than individuals with a single disorder (Galbaud Du Fort et al. 1993; Kushner et al. 2008). To avoid this bias, epidemiological data drawn from large-scale community samples can provide the most informative figures.

Over the past three decades, multiple population-based studies have surveyed the prevalence of addictive and mental disorders in the United States and abroad, including the following:

- The Epidemiological Catchment Area (ECA) survey (Regier et al. 1990) was based on diagnostic information using the *Diagnostic and Statistical Manual of Mental Disorders, Third Edition* (DSM–III) (American Psychiatric Association [APA] 1980); it was conducted between 1980 and 1984 and collected information from nearly 20,000 respondents ages 18 and older in the United States.
- The National Comorbidity Survey (NCS) (Kessler et al. 1994, 1997), also conducted in the United States, used the DSM–III–R criteria (APA 1987) while sampling 8,098 individuals ages 15 to 54 years.
- Burns and Teesson (2002) published findings on the comorbidity between AUDs and anxiety, depression, and other drug use disorders from the Australian National Survey of Mental Health and Well-Being (NSMH&WB) project. This project was a cross-sectional analysis of 10,461 Australian adults ages 18 and older, with data collected in 1997 using diagnostic criteria from the DSM–IV (APA 1994).
- The most recent epidemiological study to date, and the largest reviewed here, was the National Epidemiologic Survey on Alcohol and Related Conditions (NESARC) (Grant et al. 2004; Hasin et al. 2007). This survey, which was conducted by the National Institute on Alcohol Abuse and Alcoholism in 2001–2002, also applied DSM–IV diagnostic algorithms in a sample of 43,093 adults ages 18 and older.

The respective prevalences of comorbid anxiety disorders and AUDs from each of these epidemiological studies are summarized in table 1. These data show that, across different large-scale studies, at different times, and both in the United States and abroad, anxiety and AUDs co-occur at rates greater than would be expected by chance alone. The odds ratios (ORs) characterizing the comorbidity between an AUD and any anxiety disorder in these studies ranged between 2.1 and 3.3—in other words, the two conditions co-occurred about two to three times as often as would be expected by chance alone.

Three additional trends emerging from community-based samples are noteworthy. First, anxiety disorders are more strongly associated with alcohol dependence than with alcohol abuse (e.g., Hasin et al. 2007; Kessler et al. 1996; Kushner et al. 2008). Analysis of the NESARC data demonstrated that this finding generally was consistent across racial/ethnic groups (Smith et al. 2006). Alternative explanations for these results suggest that either people with anxiety disorders are more likely to become psychologically dependent on alcohol because they use it to self-medicate (e.g., Tran and Smith 2008) or anxiety disorders in these individuals largely are an artifact of alcohol withdrawal (e.g., Schuckit and Hesselbrock 1994).

Second, the magnitude of the relationship between specific anxiety disorders and AUDs varies across the specific combinations. For example, panic disorder typically has a relatively large association with AUDs (odds ratio [OR] = 1.7–4.1 in table 1), whereas obsessive-compulsive disorder has the least consistent and typically weakest relationship with alcohol problems (e.g., Gentil et al. 2009; Kessler et al. 1997; Schuckit et al. 1997; Torres et al. 2006). A classic review in this field (Kushner et al. 1990) indicated even more pronounced differences in the comorbidity rates of specific anxiety disorders among clinic-based samples of patients with alcohol problems. These ranged from rates near community-based rate estimates (e.g., for simple phobia) to rates nine times greater than community estimates (e.g., for social phobia). It is important to note, however, that the influence of treatment seeking and related variables confounds interpretation of these clinic-based estimates.

Third, different comorbidity patterns exist among patient subgroups with different demographic characteristics such as race/ethnicity and gender. For example, in the NESARC, Native Americans had elevated rates both of anxiety disorders and of AUDs over the past 12 months but lower rates of co-occurrence between these disorders compared with other ethnic groups (Smith et al. 2006). Gender differences in anxiety–alcohol comorbidity have been reported across a variety of samples (e.g., Hesselbrock et al. 1985; Kessler et al. 1997; Mangrum et al. 2006; Merikangas et al. 1998), and research in this area also has identified notable clinical differences between men and women. These gender differences are discussed in more detail in the sidebar.

The importance of these prevalence data is underscored by the clinical impact of comorbid anxiety and AUDs. Both types of disorder are associated with substantial societal costs that have been estimated in monetary terms at $184.6 billion per year for AUDs (Harwood 2000) and between $42 and $47 billion for anxiety disorders (DuPont et al. 1996; Greenberg et al. 1999). Kessler and Greenberg (2002) suggested that the costs for anxiety disorders were grossly underestimated and actually exceeded $100 billion per year in the total U.S. population. Furthermore, clinical studies have shown that both anxiety and AUDs can negatively impact the course and treatment outcome for the other condition. For example, anxiety problems have been associated with increased severity and persistence of AUDs, increased risk for relapse following treatment, and increased lifetime service utilization in the context of substance use disorders more generally (Driessen et al. 2001; Falk et al. 2008; Kushner et al. 2005; Johnston et al. 1991; Perkonigg et al. 2006; Sannibale and Hall 2001). Conversely, concurrent AUDs have been associated with greater severity and chronicity of anxiety disorders, and substance use problems can decrease the likelihood of recovery from anxiety disorders (Bruce et al. 2005; Hornig and McNally 1995; Schade et al. 2004). Studies also have demonstrated that alcohol use can increase anxiety (see Kushner et al. 2000), which can result in a positive feedback loop leading to exacerbation of both disorders.

Taken together, the epidemiological and clinical literature describing the relationship between anxiety and AUDs shows that this comorbidity is both prevalent and clinically relevant. Therefore, it is important to enhance understanding of this comorbidity. The following sections will review fundamental concepts related to how these disorders co-occur and describe approaches to diagnosing and treating comorbid anxiety and AUDs.

## Development of Comorbid Anxiety and AUDs

The question of how anxiety and AUDs coalesce has intrigued investigators and clinicians for decades and still is a subject of debate. Three primary pathways have been proposed:

- The common-factor model that uses a third variable to explain the co-occurrence of anxiety and AUDs;
- The self-medication pathway, which posits that people consume alcohol to cope with anxiety disorders, leading to co-occurring AUDs; and
- The substance-induced pathway, wherein AUDs lead to increased anxiety and vulnerability for co-occurring anxiety disorders.

### The Common-Factor Model

The common-factor model of comorbid anxiety and AUDs presumes that no direct causal relationship exists between the two disorders. Instead, so-called third variables are posited to account for their joint presence. The potential relevance of such factors was demonstrated in a 21-year longitudinal study of young people (Goodwin et al. 2004), in which early presence of anxiety disorders seemed to predict the later development of alcohol dependence. However, when the investigators controlled for other variables, such as prior other drug dependence and depression, the presence of anxiety disorders no longer was a significant predictor. The results of this study suggest that the link between anxiety and AUDs was not direct but instead may have been a consequence of those other variables studied. The potential range of common factors can be difficult to estimate, but a review of the literature shows that the most consistently proposed third variables are genetic factors and personality traits such as anxiety sensitivity. Support for the role of genetic factors as a cause for the co-presence of these disorders indirectly has been provided by family and twin studies (e.g., Merikangas et al. 1994, 1996; Tambs et al. 1997). Anxiety sensitivity also has been linked to the incidence of both anxiety and substance use disorders (DeHaas et al. 2001; DeMartini and Carey 2011; Schmidt et al. 2007). Based on findings demonstrating a genetic contribution to anxiety sensitivity (Stein et al. 1999), Stewart and Conrod (2008) proposed a causal sequence wherein genetic factors and anxiety sensitivity operate together to create a genetically based personality that is vulnerable to comorbid anxiety and alcohol use problems. To date, rigorous empirical evaluation of the common-factor model has been limited, and publications directly addressing this topic are sparse. Additional research and exploration of additional third variables therefore is necessary to more clearly appraise their unique and interactive influence on the relationship between these disorders.

### The Self-Medication Model

The self-medication explanation for the comorbidity of anxiety and AUDs has received the most attention in the clinical and research literature. This model proposes that people with anxiety disorders attempt to alleviate negative consequences of these conditions (i.e., are negatively reinforced) by drinking alcohol to cope with their symptoms, eventually leading to the later onset of AUDs. This concept, in fact, is shared by several models of alcoholism, including the self-medication (Khantzian 1985; Quitkin et al. 1972), tension reduction (Conger et al. 1999), and stress-response dampening models (Sher 1987; Sher and Levenson 1982). Several lines of evidence provide support for this pathway. When people with comorbid anxiety and AUDs are queried about their drinking, they typically endorse purposeful and targeted drinking to cope with their anxiety. The reported rates of self-medication in clinical samples of people with both types of disorders have ranged from 50 to 97 percent, with the highest rates among people with phobias (Bibb and Chambless 1986; Smail et al. 1984; Thomas et al. 2003; Turner et al. 1986).

It is interesting to note that participants with anxiety disorders in community samples show significantly less robust rates of self-medication than typically found in clinical samples, highlighting the potential selection bias in treatment settings (e.g., Bolton et al. 2006; Menary et al. 2011; Robinson et al. 2009). For example, in the NCS (Bolton et al. 2006) only 21.9 percent of individuals with anxiety disorders in the community endorsed self-medicating with either alcohol or drugs, with the highest rates found among those with generalized anxiety disorder (35.6 percent), panic disorder (23 percent), or social phobia–complex subtype (21.2 percent). In the NESARC, Robinson and colleagues (2009) separately analyzed rates of self-medication with alcohol, drugs, or both among respondents with anxiety disorders. The investigators found that these individuals were most likely to endorse self-medication with alcohol alone and that the highest rates of alcohol-based self-medication were found among respondents with generalized anxiety disorder (18.3 percent), social phobia (16.9 percent), and panic disorder with agoraphobia (15.0 percent). More recently published longitudinal analyses of alcohol-using NESARC participants showed nearly identical rates of self-medication with alcohol among those with anxiety disorders at both Wave 1 (20.3 percent) and Wave 2 (20.8 percent) (Menary et al. 2011). Interestingly, this report also showed that although only 1 in 5 individuals with anxiety disorders reported using alcohol to cope with anxiety, the rate of alcohol dependence in this subgroup (34.5 percent) was almost four times higher than the comparison rates found among respondents with anxiety who did not report self-medication (9.3 percent) and almost seven times higher than among respondents with no anxiety diagnosis (5.1 percent). Moreover, endorsement of alcohol-based self-medication at Wave 1 increased the risk of developing new alcohol dependence at Wave 2 nearly four-fold (OR = 3.77). These epidemiological findings reveal that although only a minority of people with anxiety disorders uses alcohol to self-medicate, the risk for co-occurring alcohol dependence is concentrated among this subgroup.

Additional epidemiological support for this causal pathway comes from analyses of order of onset as well as from analyses of whether the anxiety disorders are considered independent or substance induced. Data showing that anxiety disorders predate AUDs and that anxiety disorders are independent (i.e., not merely a consequence) of AUDs are essential prerequisites for the self-medication model. Consistent with this causal explanation of comorbidity, timelines gathered in community surveys show that anxiety disorders often predate the development of alcohol dependence. For example, Kushner and colleagues (2008) reviewed findings from several large-scale studies and calculated that three of four individuals with comorbid disorders developed the anxiety disorders first. The classification of anxiety disorders as independent versus substance-induced requires that one of two conditions is met: (1) the anxiety disorder must precede the AUD and (2) the anxiety disorder persists outside the direct influence of alcohol use. Because alcohol withdrawal can mimic and/or exacerbate anxiety problems, an extended period of abstinence (e.g., 4 weeks) from alcohol is necessary for a disorder to be considered a stand-alone, independent diagnosis. Using these criteria with the NESARC sample, which strictly followed DSM–IV rules for differential diagnosis, only 0.2 percent of anxiety disorders were not classified as independent (Grant et al. 2004). Likewise, low rates of substance-induced anxiety disorders (0.3 percent) were found in a community sample of 1,095 Australian women (Williams et al. 2010), based on DSM–IV–TR criteria (APA 2000).

Taken together, all of these findings provide compelling support for the self-medication explanation for co-occurring anxiety and AUDs. However, these lines of evidence are associated with several limitations. For example, the analyses often rely on retrospective self-reported data. Findings derived from clinical samples also can inflate prevalence estimates of self-medication, especially if alcohol-dependent individuals are evaluated during acute alcohol withdrawal. Finally, it is notable that laboratory studies examining alcohol’s anxiety-reducing (i.e., anxiolytic) effects have produced mixed findings (see Tran and Smith 2008). One possible explanation for the incongruence between laboratory and self-report survey data is that a person’s expectations about alcohol’s effects can motivate drinking independent of alcohol’s actual physiological effects (e.g., Abrams and Kushner 2004). Nevertheless, laboratory-based investigations of whether (and how) alcohol actually reduces anxiety are essential to critically evaluate the self-medication hypothesis. The current state of the science on this point is inconclusive, and additional research is necessary before any firm conclusions regarding this pathway can be drawn.

### The Substance-Induced Anxiety Model

The third causal explanation for comorbid anxiety and AUDs asserts that anxiety largely is a consequence of heavy, prolonged alcohol consumption. Alcoholism leads to a range of biopsychosocial problems, and anxiety can result from alcohol-related disturbances in each of these domains. The course of alcohol dependence is fraught with repeated intermittent episodes of excessive and frequent consumption and withdrawal, which can result in changes in the nervous systems that produce and/or worsen anxiety. For example, whereas acute alcohol intake has anxiolytic effect by increasing the activity of the brain chemical (i.e., neurotransmitter) γ-aminobutyric acid (GABA), chronic alcohol dependence results in an overall GABA deficiency that offsets the effects of acute consumption and may induce anxiety. Withdrawal periods also can induce changes in the brain, which can include excessive activity (i.e., hyperexcitability) of certain brain systems (i.e., the limbic system and the norepinephrine system) (Kushner et al. 2000; Marshall 1997), both of which are involved in the production of panic attacks (Graeff and Del-Ben 2008; Marshall 1997). Across time, repeated withdrawal episodes can result in a progressive neural adaptation (i.e., a process known as kindling) that makes the drinker more susceptible to anxiety and exacerbates stress-induced negative affect when alcohol intake stops (Breese et al. 2005). Not surprisingly, clinical studies show that people with alcoholism who are recently abstinent characteristically report increased feelings of anxiety, panic, and phobic-like behaviors in the short term, and symptoms of autonomic activity (i.e., sympathetic activation, such as increased heart rate and faster/shallower breathing) and persistent anxiety across protracted withdrawal (see Schuckit and Hesselbrock 1994).

The psychosocial impact of alcoholism also has been implicated in the genesis of anxiety. Social consequences of habitual excessive drinking are common and include pervasive and cumulative problems in vital areas of life, such as employment, interpersonal relationships, and finances (Klingemann 2001; Klingemann and Gmel 2001). In fact, such difficulties in everyday living are so intertwined with heavy use that they are reflected in the DSM–IV criteria for AUDs (APA 2000). The interaction between pathologic alcohol use and enhanced life stress can lead to anxiety in at least two ways. First, the consistent presence of social disturbances may activate and intensify anxiety symptoms among these already vulnerable individuals. Second, alcohol use in the presence of stress stimuli may interfere with extinction-based learning necessary for normal adaptation to stressors. Thus, hazardous drinking can lead to anxiety through a noxious combination of greater levels of life stress coupled with relatively poor coping skills.

**Gender Differences in Comorbid Anxiety and Alcohol Use Disorders**

Numerous studies have attempted to evaluate possible gender differences in the frequency of comorbid anxiety disorders and alcohol use disorders (AUDs). Population surveys consistently show that anxiety disorders are more common among women, whereas AUDs are more common among men (e.g., Hasin et al. 2007; Kessler et al. 1997; Lewis et al. 1996). To account for these baserate differences when estimating gender-specific comorbidity rates for anxiety disorders and AUDs in the National Comorbidity Survey, Kessler and colleagues (1997) used adjusted odds ratios (ORs). These analyses found that among alcohol-dependent men in the sample, 35.8 percent (OR = 2.22) had a co-occurring anxiety disorder, compared with 60.7 percent (OR = 3.08) among alcohol-dependent women. Moreover, not only did women in the study have an increased likelihood of independent anxiety disorders compared with men, but prior anxiety disorders also were more strongly predictive of later alcohol dependence among the women. Furthermore, a multisite trial in Germany demonstrated that anxiety disorders had a substantial influence on the course and severity of alcoholism in women (Schneider et al. 2001). Thus, in this treatment-seeking sample women who had an anxiety disorder reported an accelerated temporal sequence of alcoholism, including earlier onset of first drink, regular drinking, and incidence of alcohol withdrawal than women with no anxiety disorder.

One potential explanation for these findings is that the reasons for using alcohol may differ by gender. For example, women may be more prone than men to self-medicate for mood problems with substances such as alcohol (Brady and Randall 1999). Furthermore, empirical inspection of gender differences in stress-related drinking has shown that women report higher levels of stress and have a stronger link between stress and drinking (Rice and Van Arsdale 2010; Timko et al. 2005). Together, these results suggest that women may be more likely to rely on alcohol to manage anxiety.

Anxiety disorders also may have a particularly detrimental impact on alcohol-focused treatment for women. This has been demonstrated in a series of studies evaluating the intersection of gender, social anxiety disorder, and treatment modality. Early work in this area from the Project MATCH sample revealed an intriguing interaction (Thevos et al. 2000). Specifically, whereas socially phobic men benefitted equally well from either cognitive–behavioral therapy (CBT) or 12-step facilitation (TSF), women with social phobia fared less well if they were assigned to TSF. To shed light on the potential role of social anxiety in addiction treatment, Book and colleagues (2009) compared participants in an intensive outpatient program with high and low social anxiety on attitudes toward treatment activities. Members of the group with high social anxiety, who predominantly were female (71 percent), overall showed less treatment participation than did members of the comparison group. For example, they were less likely to speak up in group therapy, attend a 12-step meeting, or seek sponsorship within a 12-step group. A recent secondary analysis of alcoholics who were assigned to TSF in Project MATCH yielded findings consistent with and complementary to these observations, demonstrating that women with comorbid social phobia were 1.5 times more likely to relapse than noncomorbid women (Tonigan et al. 2010). In contrast, no differences in relapse rates were found among the men with or without social phobia in the study. Interestingly, socially phobic women were less likely than women without social phobia to obtain an Alcoholics Anonymous sponsor, which may help explain the poor outcomes for TSF among this subgroup.

Taken together, the findings reviewed here provide some instructive information on gender differences in the comorbidity of anxiety and AUDs. Thus, women are more likely than men to have both disorders, and the presence of anxiety disorders may exacerbate the course and severity of alcohol problems in women. Furthermore, treatment for women with this comorbidity may be especially complex, both because they are likely to use alcohol to self-medicate for stress and because women with social phobia may be reluctant to participate in treatment (e.g., Alcoholics Anonymous) that could otherwise be effective. These factors spotlight the importance of probing for anxiety disorders in women entering alcohol treatment and reinforce the need to remain sensitive to the different ways that gender can influence the process and outcomes of therapy.

ReferencesBookSWThomasSEDempseyJPSocial anxiety impacts willingness to participate in addiction treatmentAddictive Behaviors2447447620091919579410.1016/j.addbeh.2008.12.011PMC2671294BradyKTRandallCLGender differences in substance use disordersPsychiatric Clinics of North America2224125219991038593110.1016/s0193-953x(05)70074-5HasinDSStinsonFSOgburnEGrantBFPrevalence, correlates, disability, and comorbidity of DSM-IV alcohol abuse and dependence in the United States: Results from the National Epidemiologic Survey on Alcohol and Related ConditionsArchives of General Psychiatry6483084220071760681710.1001/archpsyc.64.7.830KesslerRCCrumRMWarnerLALifetime co-occurrence of DSM-III-R alcohol abuse and dependence with other psychiatric disorders in the National Comorbidity SurveyArchives of General Psychiatry,543133211997910714710.1001/archpsyc.1997.01830160031005LewisCEBucholzKKSpitznagelEShaykaJJEffects of gender and comorbidity on problem drinking in a community sampleAlcoholism: Clinical and Experimental Research20466476199610.1111/j.1530-0277.1996.tb01077.x8727239RiceKGVan ArsdaleACPerfectionism, perceived stress, drinking to cope, and alcohol-related problems among college studentsJournal of Counseling Psychology574394502010SchneiderUAltmannABaumannMComorbid anxiety and affective disorder in alcohol-dependent patients seeking treatment: The first Multicentre Study in GermanyAlcohol and Alcoholism3621922320011137325810.1093/alcalc/36.3.219ThevosAKRobertsJSThomasSERandallCLCognitive behavioral therapy delays relapse in female socially phobic alcoholicsAddictive Behaviors2533334520001089028810.1016/s0306-4603(99)00067-2TimkoCFinneyJWMoosRHThe 8-year course of alcohol abuse: Gender differences in social context and copingAlcoholism: Clinical and Experimental Research29612621200510.1097/01.alc.0000158832.07705.2215834227ToniganJSBookSWPaganoME12-Step therapy and women with and without social phobia: A study of the effectiveness of 12-step therapy to facilitate Alcoholics Anonymous engagementAlcoholism Treatment Quarterly2815116220102142356910.1080/07347321003648596PMC3060781

Evidence for the substance-induced hypothesis comes from multiple sources. A central prediction of this causal model is that abstinence from alcohol should be followed closely by a conspicuous decrement in anxiety symptoms. Data from a study of 53 patients who participated in alcohol treatment at a residential substance abuse program were consistent with this prediction (Kushner et al. 2005). Thus, among those 23 patients who had an anxiety disorder at baseline and remained abstinent after approximately 120 days, 61 percent no longer met criteria for an anxiety disorder at follow-up. Another study with 171 male veterans demonstrated that self-reported measures of temporary anxiety (i.e., state anxiety) decreased rapidly during inpatient alcohol treatment (Brown et al. 1991). It was furthermore noteworthy that scores on a measure of the participants’ overall anxiety levels (i.e., trait anxiety) also changed significantly at 3-month follow-up. This latter finding suggests that state anxiety that occurs during early abstinence can lead respondents to consider their increased anxiety levels as more chronic than they actually are. Therefore, retrospective self-reports collected at baseline should be interpreted with caution.

Additional evidence for the substance-induced pathway comes from prospective studies demonstrating that the presence of alcohol dependence predicts the later development of anxiety disorders. For example, in a sample of college students followed for 7 years, anxiety disorders increased fourfold among those diagnosed as alcohol dependent at either year 1 or year 4 of the study period (Kushner et al. 1999). A final line of support is found in differential comorbidity rates among samples of anxiety and alcohol patients. In a seminal review, Schuckit and Hesselbrock (1994) noted that the frequency of alcoholism among anxiety patients was not markedly higher than in the general population, contrary to what would be predicted by the self-medication hypothesis. In contrast, some studies have found greatly elevated rates of anxiety disorders in samples of individuals with alcohol problems (e.g., Kushner et al. 1990).

Similar to the common-factor and self-medication hypotheses, the literature underpinning the substance-induced pathway to comorbid anxiety and AUDs is convincing but cannot account for the findings consistent with the other causal models. It also is important to note that reliance on timeframes, although useful, could mask an independent course of anxiety symptoms among individuals who also have an AUD. For example, it is possible that an anxiety disorder which appears at a time when the person is experiencing alcohol-related problems may have an etiology separate from alcohol use. Likewise, a reduction in anxiety symptoms following alcohol treatment, which often is interpreted as an indication that the anxiety symptoms were a consequence of alcohol use, could also be explained by anxiolytic therapy and/or the natural course of anxiety independent of any effects related to abstinence.

Compared side by side, these proposed causal models provide competing explanations for the joint development of anxiety disorders and AUDs. It is apparent that the collective findings in this area do not unequivocally point to one pathway or exclude another. It is unclear whether this is a result of a failure of the aforementioned theoretical models or of the methods used to test the pathways or if it simply reflects the complexity inherent within this comorbidity. In fact, the support for multiple causal models may reflect that etiological differences exist among individuals who share this comorbidity, based on which disorder or predisposing variable was initially present. The continued viability of all these competing hypotheses suggests that further and more advanced research attention is essential to disentangle the predisposing factors, primary variables, sequencing, and early course involved with these co-occurring disorders.

## Mutual Maintenance of Anxiety and AUDs

Once comorbidity between anxiety disorders and AUDs has been established, the two disorders may influence and maintain each other in ways that are independent of the developmental pathway. In other words, the processes involved in the initiation and the maintenance of comorbidity may differ in meaningful ways. One hypothesis emerging from the comorbidity literature is that anxiety and AUDs become intertwined in a reciprocal, perpetuating cycle. This positive feedback loop often is characterized as a feed-forward or mutual-maintenance pattern. Stewart and Conrod (2008) dubbed this progressive sequence the “vicious cycle of comorbidity” in which biopsychosocial outcomes of one disorder (e.g., anxiety) serve to maintain or even worsen the other disorder (e.g., alcoholism), whose respective outcomes, in turn, further maintain or exacerbate the first disorder, and so on. For example, a person who copes with anxiety by self-medicating with increasing amounts of alcohol likely will experience greater alcohol-related consequences (e.g., poor job performance, interpersonal problems, and anxiety induction from alcohol withdrawal), thus exacerbating the initial anxiety and leading to further drinking, which in turn sustains and/or amplifies the cycle.

Empirical support for this mutual-maintenance model comes from various sources, which in many ways reflects a synthesis of data supporting the three developmental pathways. Taken together, the sets of supportive findings suggest that (1) anxiety disorders can increase the severity, persistence, and poor treatment response of comorbid AUDs and (2) AUDs can increase the severity, persistence, and poor treatment response of comorbid anxiety disorders. Evidence that comorbid anxiety disorders can worsen and perpetuate AUDs and impair alcohol treatment response includes the following findings:

- People with social anxiety disorder endorsed greater alcohol dependence severity and had more dependence symptoms than alcoholics without social phobia (Thomas et al. 1999).
- The presence of social anxiety disorder and generalized anxiety disorder predicted increased long-term mental distress among treatment-seeking, substance-dependent patients (Bakken et al. 2007).
- Alcoholic inpatients with anxiety disorders had increased severity of alcohol withdrawal (Johnston et al. 1991).
- Comorbid panic disorder with agoraphobia and generalized anxiety disorder were related to increased risk of persistent alcohol dependence (Falk et al. 2008).
- Symptoms of generalized anxiety disorder and social anxiety disorder can interfere with substance use treatment (Book et al. 2009, Smith and Book 2010).
- Anxiety disorders are associated with elevated risk for relapse following alcohol treatment (e.g., Driessen et al. 2001; Kushner et al. 2005).

Similarly, other studies reported a negative impact of comorbid AUDs on the course of anxiety disorders, consistent with the mutual maintenance hypothesis, as follows:

- AUDs were related to increased psychiatric severity among individuals who were diagnosed with phobic disorders (Schade et al. 2004).
- People with panic disorder who also had a substance use disorder were significantly more likely to report attempted suicide (Hornig and McNally 1995).
- Repeated withdrawals from alcohol can produce neurobiological changes that sensitize anxiety (Breese et al. 2005).
- Substance use disorders were associated with chronicity of generalized anxiety disorder (Bruce et al. 2005).
- Substance use disorders predicted worse outcomes following treatment for patients with panic disorder with agoraphobia, generalized anxiety disorder, and social anxiety disorder (Bruce et al. 2005).

Collectively, these independent findings are consistent with the mutual-maintenance model of comorbid anxiety and AUDs. However, although it may be reasonable to infer that the pattern of results demonstrates the heuristic utility of this model as a way to synthesize outcomes from various studies in this research area, the conclusion that a discontinuity between developmental and maintenance phases of this comorbidity exists remains speculative. Furthermore, to date no studies have empirically tested these dynamic and interactive factors in a longitudinal model. Thus, the status of the science underpinning the mutual maintenance hypothesis at this time only yields indirect agreement.

## Diagnostic and Treatment Considerations for Comorbid Anxiety and AUDs

The developmental and maintenance factors associated with comorbid anxiety and AUDs show that the pairing of these two types of disorders is heterogeneous, interactive, and potentially progressive. Treatment approaches for comorbid patients correspondingly require comprehensive assessment and thoughtful planning. One paramount concern is the establishment of the correct diagnosis and exclusion of other diagnoses, especially because of the inherent difficulty in discerning whether anxiety present at the initial assessment is substance-induced or the sign of an independent anxiety disorder. As indicated earlier, reliance on self-report data can impair the accuracy of diagnoses, especially in the presence of recall bias that can be expected when a person is acutely anxious (e.g., Brown et al. 1991). Careful assessment therefore entails gathering a thorough and detailed retrospective timeline, interviewing collateral informants, reviewing the patient’s medical record and any available laboratory data, and observing symptoms over a sustained period of abstinence (Anthenelli 1997; Watkins et al. 2005). The exact duration of abstinence necessary to establish an independent anxiety disorder varies across disorders. For example, anxiety disorders whose cardinal symptoms are consistent with anxiety induced by alcohol withdrawal (e.g., panic disorder and generalized anxiety disorder) require longer periods of abstinence for a diagnosis than anxiety disorders with less symptom overlap (e.g., obsessive-compulsive disorder). Thus, a prudent diagnostician will wait several weeks to determine the likely source of symptoms that also frequently occur during withdrawal, such as panic or free-floating worry. Conversely, certain types of anxiety (e.g., social anxiety) typically predate alcohol use problems, and the presence of these symptoms therefore is less likely to be an artifact of alcohol withdrawal. A more comprehensive diagnostic algorithm for differential diagnosis is provided by Anthenelli (1997). A realistic limitation of the diagnostic process is that some individuals may not be able to sustain abstinence for a period long enough to clarify whether the constellation of anxiety symptoms represents a substance-induced syndrome or an independent anxiety disorder. In such cases, a prospective functional analysis may be used to identify the antecedents and consequences of both anxiety and alcohol use (Wyman and Castle 2006).

Perhaps most importantly, once the complete assessment data have been gathered through all the available strategies, the full spectrum of information should be integrated and considered as a whole to yield the most accurate diagnosis. To select an appropriate treatment approach using these differential diagnosis methods it also is crucial to consider that substance-induced mood and anxiety disorders can negatively impact treatment and increase overall clinical severity (Grant et al. 2004). Consequently, when it has been determined that an anxiety disorder likely is substance induced it may not be the best approach to simply treat the AUD alone and wait for the subsequent remission of the anxiety disorder.

When a diagnosis has been established, the treatment provider also needs to take into consideration the unique factors associated with this comorbidity when selecting the appropriate treatment protocol. As discussed below, a variety of pharmacotherapy and psychotherapy approaches are available to address anxiety and AUDs. Each modality has proven to be efficacious for these problems in isolation, and several evidence-based treatment alternatives for each disorder are available (see table 2). However, it sometimes may be necessary to modify these treatment approaches for comorbid individuals because even strategies considered the gold standard for one disorder potentially can have a negative impact on individuals with the other disorder (e.g., Jenson et al. 1990; Larson et al. 1992; Randall et al. 2001; Thevos et al. 2000; Tonigan et al. 2010).

### Pharmacotherapy for Anxiety Disorders

Medication-based treatments for anxiety include an assortment of agents from several classes of medication, including benzodiazepines, tricyclic antidepressant drugs (TCAs), monoamine oxidase inhibitors (MAO-Is), and serotonergic-based medications (e.g., selective serotonin reuptake inhibitors [SSRIs], serotonin-norepinephrine reuptake inhibitors [SNRIs], and the 5-HT_1a_ partial agonist buspirone). The efficacy of these drugs for anxiety treatment has been established firmly in well-controlled, randomized clinical trials. However, it is important to note that these studies typically exclude people with AUDs—a requisite standard practice to enhance the internal validity of efficacy studies. This exclusion means, however, that treatment providers must use clinical judgment when prescribing these medications to comorbid patients.

#### Benzodiazepines

Benzodiazepines can be very safe and effective agents for the short-term management of anxiety disorders. These medications are well-tolerated and have few medical scenarios in which they must not be used (i.e., few contraindications), although patients with pulmonary disorders may be sensitive to the depressant effects of these agents on the central nervous system. Because these medications are absorbed into the body fairly rapidly, patients can experience relatively fast-acting anxiolytic effects from a single oral dose. When multiple doses of benzodiazepines are used to manage anxiety, the duration of action will vary based on the medication’s accumulation in the body, which is determined by pharmacokinetic characteristics such as elimination half-life and clearance. According to their elimination half-life, benzodiazepines can be classified into three groups (Greenblatt et al. 1981):

- Ultra–short-acting agents with a half-life of less than 5 hours (e.g., triazolam, midazolam);
- Intermediate/short-acting agents with a half-life of 5 to 24 hours (e.g., alprazolam, lorazepam); and
- Long-acting agents with a half-life of more than 24 hours (e.g., clonazepam, diazepam).

Because benzodiazepines are effective in managing anxiety in the short-term by producing a relatively fast-acting anxiolytic effect, their use as a front-line choice for individuals with comorbid anxiety and AUDs has been controversial (e.g., Brady and Verduin 2005; Ciraulo and Nace 2000; Posternak and Mueller 2001; Sattar and Bhatia 2003). For example, when discussing the relative benefits and risks associated with these medications, Longo and Johnson (2000) elegantly stated that, “Their greatest asset is also their greatest liability: drugs that work immediately tend to be addictive.” (p. 2127). Perhaps not surprisingly, the addiction potential of benzodiazepines is highest for the shorter-acting compounds as well as for those agents (e.g., alprazolam) that quickly cross the blood–brain barrier (Longo 1998; Martinez-Cano et al. 1996; Roache and Meisch 1995). People who have a history of AUDs seem to be more sensitive to the rewarding properties of these agents, and benzodiazepines have a positive effect on mood in alcoholics that is not seen in nonalcoholics (Ciraulo et al. 1988, 1997). Additional findings from clinical samples alternately have shown that abuse of sedatives (mostly benzodiazepines) among patients with anxiety was associated with concurrent alcoholism (Van Valkenberg 1999) and that alcohol-dependent patients (who also engaged in other drug abuse) were more likely to abuse benzodiazepines if they also reported panic attacks (Jenson et al. 1990). These factors together suggest an enhanced risk of benzodiazepine misuse among people with co-occurring anxiety and AUDs. Because effective and safe alternatives to manage anxiety are available (e.g., SSRIs and buspirone), it has been suggested that because of these risks, benzodiazepines generally should be avoided when treating patients with alcoholism, especially those with severe alcohol dependence or polydrug abuse (e.g., Longo and Bohn 2001; Sellers et al. 1993).

Some clinical scholars have questioned this viewpoint, however, and proposed that withholding access to potentially beneficial medications is unethical, especially when some studies suggest that a history of substance abuse is not a major risk factor for benzodiazepine abuse (e.g., Posternak and Mueller 2001; Sattar and Bhatia 2003). For example, in prospective studies Mueller and colleagues (1996, 2005) found little evidence that these anxiolytics were associated with poor outcomes among those with both anxiety and AUDs.^1^ Specifically, they found that (1) a history of AUDs was not a strong predictor of benzodiazepine use among participants with anxiety disorders, (2) use of these anxiolytics did not increase across time among comorbid participants, and (3) benzodiazepine use was not associated with the later occurrence of any new AUDs. These findings suggest that although the risk for benzodiazepine abuse should be an important consideration when prescribing within this patient subpopulation, these agents safely may be used in cases where they are clinically indicated (e.g., when other treatments are ineffective or potentially harmful). When benzodiazepines are used, patients should be monitored closely and only limited amounts of the agents should be prescribed. A useful algorithm to guide treatment decisions for people with co-occurring anxiety and AUDs was provided by Sattar and Bhatia (2003).

#### MAO-Is and TCAs

Caution also is suggested with the use of MAO-Is and TCAs for comorbid individuals. Although MAO-Is are quite effective in reducing anxiety, patients taking these agents may suffer a sudden severe increase in blood pressure (i.e., hypertensive crisis) after consuming certain foods and beverages that contain the amino acid tyramine (McCabe-Sellers et al. 2006), resulting in dietary restrictions for MAO-I users. These beverages include certain beers (e.g., imported beers, beer on tap, and nonalcoholic or reduced-alcohol beers), red wines, sherry, liqueurs, and vermouth, which is critical to know when treating people who also have alcohol problems. TCAs also should be used with caution among people with co-occurring AUDs and be prescribed only after other treatments have been ruled out because these medications can have an enhanced adverse-effect profile in this population. Moreover, the impaired judgment and impulsivity among persons with co-occurring alcohol use problems may increase the risks of taking an overdose of the medications that can result in toxicity and, potentially, suicidality. Finally, TCAs may react with alcohol in the brain to cause respiratory depression (Bakker et al. 2002).

#### Serotonergic-Based Medications

Medications that target a brain signaling system which uses the neurotransmitter serotonin and its receptors perhaps are the safest and most widely used agents to treat anxiety disorders. These agents include the SSRIs, SNRIs, and the serotonin partial agonist buspirone. At present, SSRIs (e.g., fluoxetine, paroxetine, and sertraline) and SNRIs (e.g., venlafaxine and duloxetine) generally are used as first-line treatment in this area because they consistently demonstrate anxiolytic efficacy, including in patients with comorbid AUDs. For example, a direct examination of the efficacy of paroxetine in this population showed that it reduced social anxiety relative to placebo (Book et al. 2008), providing an empirical foundation for its use in these patients. Moreover, serotonergic agents have favorable properties, such as being well-tolerated and having virtually no abuse potential. Another welcome characteristic of SSRIs in patients with comorbid AUDs is that, in contrast to TCAs, they do not interact with alcohol to increase the risk of respiratory depression (Bakker et al. 2002). With both SSRIs and SNRIs it is advisable to inform patients that it may take about 1 to 2 weeks before these medications show full effectiveness. In addition, there is a risk of an electrolyte imbalance involving decreased sodium concentrations in the blood (i.e., hyponatremia), which can reduce the seizure threshold. This may be especially relevant during alcohol withdrawal, and clinicians therefore should monitor fluid intake and sodium levels during these periods.

Buspirone specifically is approved by the U.S. Food and Drug Adminstration (FDA) for the management of generalized anxiety disorder. Similar to other serotonergic-based medications, buspirone has a desirable safety profile but a relatively delayed onset of anxiolytic effects. Previous trials have evaluated buspirone among patients with comorbid generalized anxiety disorder (or anxiety symptoms) and AUDs. The majority of these studies have found reductions in both anxiety and alcohol outcome measures, including cravings (Bruno 1989; Tollefson et al. 1991) and drinking measures (Kranzler et al. 1994). However, one study found no effect of buspirone on either anxiety or alcohol use (Malcolm et al. 1992).

### Psychotherapy for Anxiety Disorders

The psychosocial treatment of choice for anxiety disorders is established more clearly, with a family of strategies known collectively as cognitive–behavioral therapies (CBTs) considered the practice standard for people with anxiety problems. Meta-analyses of CBTs for anxiety disorders have shown strong evidence for their efficacy (Hofmann and Smits 2008; Olatunji et al. 2010). The CBT approaches to anxiety consist of two overarching strategies (Gerardi et al. 2009):

- Exposure to feared stimuli; and
- Anxiety management techniques, such as cognitive restructuring, applied relaxation, and coping skills training.

Exposure to feared stimuli is a powerful and active treatment ingredient that is recommended across the spectrum of anxiety disorders. Although the specific cues differ, application of exposure for each disorder generally involves repeated presentation of feared stimuli until the patient has become used to them (i.e., habituation is reached), resulting in extinction of the fear response. The technique largely is effective because when clients who typically avoid and/or escape from situations that lead to anxiety are exposed to these situations for prolonged periods, they encounter corrective information that previously was unavailable.

It nevertheless is appropriate to recognize that anxious clients who also have comorbid AUDs may be vulnerable to negative outcomes from this treatment method. For many of these individuals, drinking itself is a means of limiting exposure to feared situations and thus can be conceptualized as an avoidance strategy that has prevented the development of alternative ways of coping. To borrow terminology from the respective CBT approaches for anxiety and AUDs, the link between anxiety and drinking for comorbid clients may mean that in effect an exposure exercise also becomes a high-risk situation for alcohol relapse. Relapse to avoidance strategies (e.g., reliance on checking behaviors in obsessive-compulsive disorder or avoidance of social gatherings in social anxiety disorder) in the process of exposure is undesirable even for people suffering only from an anxiety disorder. For people who use alcohol as an avoidance strategy, however, a relapse can be especially costly. Moreover, use of alcohol to avoid anxiety during an exposure exercise also can interfere with the corrective learning process required for extinction of the anxiety response. Indeed, research findings suggest that exposure-based methods can lead to worse alcohol outcomes for comorbid individuals and that alcohol use during exposure may hinder extinction (e.g., Randall et al. 2001). Therefore, as a matter of course clinicians carefully should appraise this risk when weighing the potential costs and benefits of this CBT component for people with comorbid anxiety and AUDs. To address this issue, treatment providers may try to enhance the clients’ preparedness by focusing on relapse prevention skills prior to engaging in exposure exercises, especially those activities requiring the direct confrontation of feared stimuli (e.g., during prolonged in vivo exposure therapy). Also, therapists can manage the intensity of exposure therapy by introducing clients to feared stimuli using intermediate or purposefully protracted techniques, such as imaginal exposure (e.g., retelling traumatic memories or imagining feared situations or objects) and graded exposure (e.g., step-by-step exposure to stimuli based on a fear hierarchy). Such alterations can allow therapists to calibrate the dose of exposure that optimizes efficacy for extinction of the target fear response while minimizing the risk for relapse to drinking.

#### Pharmacotherapy for AUDs

There currently are three medications that have received FDA approval for the maintenance treatment of alcoholism:

- Disulfiram, an agent that interferes with ethanol metabolism and induces an adverse reaction (e.g., flushing, nausea, and rapid heartbeat) when a person consumes alcohol;
- Naltrexone, an antagonist acting at receptors for signaling molecules, endogenous opioids, that can interfere with the rewarding properties of alcohol and reduce craving; it is available in both short- and long-acting formulations; and
- Acamprosate, an agent that acts on the GABA system, counteracting alcohol’s effects on this system.

Another drug receiving strong empirical support for the treatment of alcohol dependence is the anticonvulsant topiramate (Shinn and Greenfield 2010), although its use has not yet been approved by the FDA. Topiramate reduces the release of the neurotransmitter dopamine in the midbrain, which may reduce the rewarding experiences associated with alcohol intake. However, it is unclear at this time whether adverse effects may hinder its utility as an adjunctive alcoholism treatment, because a recent review of 26 published studies found that its use was associated with high rates of numbness of tingling on the skin (i.e., paresthesia) and cognitive symptoms (Shinn and Greenfield 2010). Additional research in randomized trials evaluating topiramate alongside more established medications, such as disulfiram and naltrexone, may shed light on its relative efficacy and tolerability.

Administration of medications for AUDs may require some adjustment for individuals who also have anxiety disorders compared with the regimen for alcoholics without this comorbidity. As with other conditions, randomized, controlled trials of pharmacotherapies to determine efficacy for alcoholism treatment often exclude individuals with comorbid conditions. Therefore, the impact of these agents on co-occurring psychiatric symptoms has not been explored fully. Some early clinical reports have suggested that disulfiram may precipitate psychiatric problems such as anxiety (e.g., Larson et al. 1992; Snyder and Keeler 1981). However, more thorough analyses suggest that these reports may not reflect current conceptualizations of psychiatric symptoms and dosing schedules (see Petrakis et al. 2002). Another concern is that people with a comorbid anxiety disorder may be taking additional medications to treat their concurrent condition and clinicians therefore must remain vigilant of potential interactions and dosage scheduling associated with multiple drugs. In one study in this underexamined area, data from 254 individuals treated for alcohol dependence on an outpatient basis and with other comorbid psychiatric disorders (including generalized anxiety disorder and panic disorder) showed that both naltrexone and disulfiram were effective and well-tolerated in this population (Petrakis et al. 2005). And in a secondary analysis of a randomized, double-blind trial Krystal and colleagues (2008) reported that among patients receiving antidepressants for mood/anxiety disorders, those receiving naltrexone showed greater reductions in drinking than did those receiving a placebo. Nevertheless, at least in the case of disulfiram, the combination of some historical clinical reports of anxiety induction and overall limited data suggests that clinicians administering this medication should closely monitor comorbid patients for any signs of increased anxiety.

### Psychotherapy for AUDs

Psychosocial approaches to treating AUDs have evolved markedly over the past few decades. The historical roots of this treatment modality largely can be traced back to the development of Alcoholics Anonymous (AA) in Akron, Ohio, in the 1930s and 1940s. It has been estimated that nearly 1 in every 10 Americans has attended at least one AA meeting, and it is “the most frequently consulted source of help for drinking problems” (McCrady and Miller 1993, p. 3). Anecdotal and research evidence suggests that AA participation can promote positive alcohol-related outcomes (e.g., Project MATCH Research Group 1997, 1998; McKellar et al. 2003; Tonigan 2009), lending some credence to the oft-quoted adage, “It works if you work it.” Several alternative treatments have been developed since and have received favorable empirical support. In a systematic analysis of 10 published reviews of evidence-based psychosocial therapies for AUDs, a majority of the reviews found support for CBTs, the community reinforcement approach (CRA), motivational interviewing (MI), relapse prevention therapy (RPT), social skills training (SST), behavioral marital (couples) therapy (BCT), and brief intervention (BI) (Miller et al. 2005).

Similar to the other modalities described here, administration of these psychosocial treatment strategies for alcohol problems can be less straightforward with individuals who have comorbid anxiety and AUDs. Clients with social anxiety disorder, for example, may have difficulties with several elements of standard psychosocial approaches for alcoholism. Many treatment programs, as well as AA, heavily rely on the mutual help in group settings. Individuals with social anxiety, however, may be reluctant to attend group therapy or AA meetings or may avoid meaningful participation should they make the effort to attend. Other activities that are integral to participation in AA, such as sharing one’s story (i.e., public speaking), obtaining a sponsor, and becoming a sponsor (i.e., initiating social contact) also can be impaired among socially anxious alcoholics. Consistent with these hypotheses, research has shown that at least among women with social phobia, participation in AA may be less appealing and less effective than other approaches (Thevos et al. 2000; Tonigan et al. 2010). Two critical elements of CBT skills training also may be especially difficult for patients with comorbid social anxiety disorder, including drink-refusal skills and enhancing one’s social support network. In essence, clients need to show assertiveness to engage in the parallel process of ending relationships and habits that are high risk for relapse while also proactively initiating contacts and improving relationships with others who will support recovery efforts. Therefore, clients in CBT who also have social anxiety may particularly benefit from additional practice with assertiveness, perhaps including adjunctive social-skills training.

Standard delivery of RPT also may require a pivotal adaptation when applied to clients with comorbid anxiety disorders. RPT emphasizes the importance of identifying an individual’s unique risk factors (e.g., high-risk situations) for relapse and incorporates skill-development techniques to help reduce the likelihood of lapses and to manage them should they occur. It is widely understood in the RPT literature that negative emotional states are particularly perilous to recovery efforts. A classic analysis of over 300 relapse episodes implicated negative emotional states, conflict with others, and social pressure to use in nearly 75 percent of the relapses studied (Cummings et al. 1980). To prevent relapse resulting from negative emotional states such as anxiety, RPT recommends stimulus control (i.e., avoidance of high-risk situations, with escape as the next best option) as a first-order strategy (Parks et al. 2004). Relaxation training also is recommended because it “can help clients reduce their anxiety and tension when facing stressful situations and minimize their typical levels of motor and psychological tension” (Parks et al. 2004, p. 78). For clients with both alcohol use and anxiety disorders, however, a potential limitation of RPT is that avoidance of anxiety-inducing situations can preclude any potential anxiety reduction via exposure therapy, which in contrast requires clients to directly confront such situations. In short, for comorbid individuals, the avoidance and escape-oriented coping strategies taught within RPT could perpetuate anxiety problems. Skillful use of RPT with this subgroup of alcoholics therefore may require adjustments to complement the goals of exposure therapy for anxiety (e.g., allowing prolonged in vivo exposure within carefully planned high-risk situations designed to elicit anxiety) while also reducing the chances of drinking as much as feasible. This can be achieved, for example, by using abstinence-focused social support during in vivo exposure to situations eliciting anxiety or by conducting in vivo exposure only in environments without access to alcohol. A structured plan using imaginal and/or graded exposure to cues that elicit anxiety also may offer a practical balance of therapeutic risk and reward.

It also is notable that comorbid individuals seem to be especially ambivalent about changing their alcohol use (e.g., Grothues et al. 2005; Velasquez et al. 1999). For example, Grothues and colleagues (2005) found that people with problematic drinking and a comorbid anxiety disorder were more likely to be in the contemplation stage of change compared with problematic drinkers with or without depression, that comorbid participants rated both the positive and negative aspects of drinking higher than comparison groups, and that they had lower self-efficacy to quit drinking. Also, both Grothues and colleagues (2005) and Velasquez and colleagues (1999) found that comorbid individuals reported greater temptation to drink than did individuals without comorbidity. People who are highly ambivalent regarding their desire to stop drinking characteristically experience two opposing alcohol-related motivations—the desire to experience the pleasure associated with drinking (i.e., an appetitive-approach motivation) and the desire to avoid alcohol and its negative consequences (i.e., negative-avoidance motivation). This ambivalence can be a negative prognostic indicator. For example, profiles of approach–avoidance drinkers have discriminated between “high lapsers” and abstainers among alcohol-dependent patients (Stritzke et al. 2007). These findings jointly suggest that ambivalence about changing alcohol use may be particularly salient among people with comorbid anxiety and AUDs, such that decisional balance likely is a principal treatment target.

The resolution of such ambivalence is a key concept of MI and is considered essential for a meaningful change to occur (Miller and Rollnick 1991, 2002). Accordingly, this counseling style seeks to help clients resolve their ambivalence by eliciting a specific class of verbal expressions (i.e., change talk) within sessions that most strongly are associated with actual behavior changes, especially phrases that signify a desire, ability, reasons, need, commitment, or steps taken to reach specified goals (Rollnick et al. 2007). An MI approach therefore may be particularly well-suited for clients with high ambivalence. In fact, meta-analyses have provided support for MI as a BI for problem drinking (Vasilaki et al. 2006). However, brief MI may not be optimal for drinkers with comorbid anxiety disorders because previous studies reported no additive benefit of BIs on either drinking outcomes or further help-seeking in this dually diagnosed population (Grothues et al. 2008*a*, *b*).

### Application of Treatment Methods

In addition to adjusting standard pharmacotherapy and psychotherapy protocols for anxiety and AUDs when treating comorbid clients, it also is crucial to apply these methods in a way that produces the best outcomes for both disorders. Case conceptualizations that implicate one disorder as primary (e.g., because the patient histories are consistent with either the self-medication or the substance-induced models of comorbidity development) may tempt clinicians to focus treatment solely on that primary disorder. However, it generally is accepted in the comorbidity literature that this approach is not advisable (e.g., Kushner et al. 2007; Lingford-Hughes et al. 2002; Stewart and Conrod 2008). As reviewed earlier, one implication of the mutual-maintenance model of comorbidity is that neglecting to treat the second disorder would place individuals at high risk of relapse to the disorder that was treated, and published studies have supported this notion (e.g., Bruce et al. 2005; Driessen et al. 2001; Kushner et al. 2005). Recommendations to treat both anxiety and AUDs therefore appear warranted on both theoretical and empirical grounds. The literature for treating dual problem specifies three primary approaches, including the sequential, parallel, and integrated models (for a comparison, see table 3).

#### The Sequential Approach

In the sequential approach to treating comorbid anxiety and AUDs one disorder is treated prior to addressing the other disorder. Advocates of this approach point out that it may be prudent to begin, for example, by treating a client’s alcohol problem and waiting to see whether abstinence leads to remission of the psychiatric problem (e.g., Allan et al. 2002; Schuckit and Monteiro 1988). This model also allows clinicians to engage clients who may be more ready to address one disorder than the other, and this may be a pragmatic early treatment strategy for comorbid clients who may only have interest in changing one of their problems (Stewart and Conrod 2008). This hypothesis is supported by recent findings from a double-blind, randomized controlled trial of paroxetine for comorbid social anxiety and AUDs, which demonstrated that although this medication did not modify drinking overall, it did reduce drinking prior to social situations and appeared to uncouple social anxiety and alcohol use (Thomas et al. 2008). The results of this study suggest that paroxetine may be useful in this subgroup of alcoholics by alleviating social anxiety as a reason for drinking, and that once social anxiety symptoms are reduced, the stage may be set for the introduction of an alcohol intervention. Examination of this sequential treatment strategy is underway.

#### The Parallel Approach

The parallel-treatment approach requires that specific treatments for both disorders are delivered simultaneously, although not necessarily by the same provider or even in the same facility. However, coordination among providers and between facilities becomes a critical issue with parallel treatments when they are not colocated. There are noteworthy advantages of this approach relative to sequenced treatment, such as, at least theoretically, reducing the chances of relapse by attending to both disorders. In light of the mutual-maintenance patterns mentioned earlier this may be a quite significant benefit. Also, parallel treatment may be sensible from a practical standpoint, given that in the current treatment culture addiction and mental health settings generally are separated and efforts to unify and integrate treatment services for comorbid clients have lagged well beyond expert recommendations (Substance Abuse and Mental Health Administration [SAMHSA] 2002). However, several limitations of the parallel approach also exist beyond inherent difficulties with case coordination (Stewart and Conrod 2008). For example, clients may become overburdened with the time and effort involved with participation in two treatments with potentially two providers in separate locations. Thus, previous research has suggested that parallel psychosocial treatments for anxiety and AUDs may be too demanding for clients, which can negatively influence treatment outcomes (Randall et al. 2001). In addition, the parallel approach may convey an implicit (and erroneous) suggestion that the two disorders are separate, and the approach generally may be inefficient.

#### The Integrated Approach

Integrated treatment strategies are akin to parallel methods of combining treatments, but with two additional features: both disorders are treated by a single provider and treatment explicitly addresses the functional interrelationship of the comorbid disorders. This intuitively appealing approach theoretically is matched to the mutual maintenance model, is efficient, and communicates to clients that their dual problems are in fact intertwined and equally require management. Based on the range of potential advantages associated with integrated therapy, expert opinion strongly suggests adopting this approach to treating anxiety and AUDs (e.g., Castle 2008; Stewart and Conrod 2008; Watkins et al. 2005). Research has provided some support for such an integrated approach in the case of co-occurring panic disorder and AUDs (Kushner et al. 2006, 2009). To date, however, unfortunately only few data exist on integrated treatment, and the incongruence between the strength of expert opinion and paucity of supportive data has been noted in several reviews (Baillie et al. 2010; Hesse 2009; Smith and Book 2008; Stewart and Conrod 2008; Watkins et al. 2005). In addition, the practical obstacles to achieving integrated treatments also are considerable, including the need for specialty training in an underdeveloped area, conceptual incongruence between elements of standard anxiety and AUD treatments, and relative lack of funding opportunities from granting agencies for these niche treatments.

Because of the overall lack of empirical data to guide clinical decisions on how to best sequence and combine therapies for anxiety disorders and AUDs, it is recommended that clinicians consider and weigh the relative advantages and disadvantages of each approach when planning treatment for their patients. The sequential, parallel, and integrated models each are beneficial in certain respects, and each method should be considered a valuable option in the practitioner’s toolkit.

## Summary and Conclusions

The comorbidity of anxiety disorders and AUDs is fairly prevalent and clinically relevant. A growing body of literature has illuminated the developmental pathways through which these disorders merge, including the common factor, self-medication, and substance-induced routes. Although epidemiological evidence most strongly supports the self-medication pathway, empirical support exists for each of these competing models, suggesting that this comorbidity is heterogeneous in its origin. Regardless of the method of onset, however, once anxiety and AUDs co-occur, the mutual maintenance model suggests that these comorbid disorders can become engaged in a feed-forward cycle that could be progressive if left untreated. It is important to be mindful of the unique developmental and maintenance characteristics associated with this comorbidity, because these elements have a considerable influence on both diagnosis and treatment planning.

Fortunately, several evidence-based strategies are available for treating anxiety and AUDs, including both pharmacotherapy and psychotherapy approaches. Administration of these methods for comorbid individuals is complex and may require modification of standard procedures to yield the greatest efficacy. It also is notable that the optimal sequence and timing of treatments remain undetermined even after decades of scientific inquiry. Although it generally has been accepted that both the anxiety disorders and the AUDs should be treated and that integrated approaches should produce the best outcomes, data on the efficacy of combined treatment approaches are limited in scope and mixed overall (e.g., Baillie et al. 2010; Schade et al. 2003; Watkins et al. 2005). In light of the current evidence, the most practical approach to combining treatments is to weigh the benefits and drawbacks of each method and apply them judiciously.

Additional advances and expansion of the empirical evidence are necessary to further move this area of research and clinical practice forward. The significant impact of empirical evidence already is evident when reflecting on the evolution of expert opinion regarding the development and treatment of comorbid anxiety and AUDs. Although these issues likely will not be settled unequivocally, recent epidemiological studies have shown that anxiety disorders among alcoholics often are independent (e.g., Grant et al. 2004; Williams et al. 2010) and clinical studies have demonstrated that efficacious treatment of one disorder does not necessarily yield improvements in the untreated comorbid disorder (e.g., Thomas et al. 2008). Together, these lines of research support putative recommendations that both disorders should be treated (see Castle 2008; Smith and Book 2008; Stewart and Conrod 2008; Watkins et al. 2005). This understanding and standard of care is a significant departure from earlier views that anxiety in this population mainly was a residual effect of heavy alcohol use and would subside with abstinence. Despite the significant contributions that have led to this paradigm shift, the anxiety–alcohol literature has reached a plateau that is defined by frequent reviews but relatively limited original research, especially in the area of randomized clinical trials with comorbid participants as the defined population of study. A practical limitation for such studies is that many potential anxiety disorder–AUD combinations exist, and developing evidence-based protocols for each combination would require a significant investment of resources. Future work may circumvent this difficulty if the recent emergence of transdiagnostic approaches to treating anxiety disorders (Norton and Philipp 2008) generates interventions that are effective across the anxiety spectrum. Transdiagnostic approaches to anxiety treatment focus on common clinical features and maintaining processes among the anxiety disorders, and are designed to synthesize evidence-based components of anxiety disorder treatments into a unified program. This innovative development would open the door to new lines of research primed to produce significant advances in the field. For example, such research could examine which shared features of anxiety disorders are associated with alcohol-related problems and whether a universal evidence-based transdiagnostic anxiety–AUD treatment protocol focused on these factors could be achieved rather than requiring separate evidence-based treatments for each anxiety disorder–AUD combination. As these and other lines of research in comorbid anxiety and AUDs continue to mature, future studies should provide further insights into the special considerations, treatment needs, and ideal therapeutic strategies for individuals with these dual problems.

## Figures and Tables

**Table 1:** Adjusted Odds Ratios of the 12-Month Comorbidity Between Certain Anxiety Disorders and Alcohol Use Disorders Across Epidemiological Samples

	**ECA**	**NCS**	**NSMH & WB**	**NESARC**
Agoraphobia	2.7	2.6	2.3	3.6
Generalized anxiety disorder	—	4.6	3.3	3.0
Obsessive–compulsive disorder	—	—	2.7	—
Panic disorder	4.1	1.7	3.9	3.5
Simple phobia	2.0	2.2	—	2.3
Social phobia	1.8	2.8	3.2	2.3
**Any**	**2.1**	**2.6**	**3.3**	**2.7**

NOTES: ECA = Epidemiologic Catchment Area Survey; NCS = National Comorbidity Survey; NSMH & WB = National Survey of Mental Health & Well-being; NESARC = National Epidemiologic Survey on Alcohol and Related Conditions.

**Table 2:** U.S. Food and Drug Administration (FDA)-Approved and Evidence-Based Treatments for Anxiety and Alcohol Use Disorders^a,b,c^

	**Generalized Anxiety Disorder**	**Obsessive–Compulsive Disorder**	**Panic Disorder**	**Social Anxiety Disorder**	**Alcohol Use Disorders**
**Pharmacotherapy**	Buspirone Duloxetine Escitalopram Paroxetine Venlafaxine	Clomipramine Fluoxetine Fluvoxamine Paroxetine Sertraline	Alprazolam Clonazepam Fluoxetine Paroxetine Sertraline Venlafaxine	Fluvoxamine Paroxetine Sertraline Venlafaxine	Acamprosate Disulfiram Naltrexone Topiramate
**Psychotherapy**	Cognitive and behavioral therapies	Cognitive therapy; exposure and response prevention	Applied relaxation; cognitive and behavioral therapies; psychoanalytic therapy	Cognitive and behavioral therapies	Behavioral couples therapy; brief intervention; cognitive and behavioral therapies; community reinforcement approach; motivational interviewing; relapse prevention therapy; social skills training; 12-step facilitation

NOTES:

a Pharmacotherapies listed are current FDA-approved indications, with the exception of topiramate, which was added based on results of a critical review of published literature (Shinn and Greenfield 2010).

b Psychotherapies for anxiety disorders are those with moderate or strong research support, as listed by the American Psychological Association, Division 12 (Society of Clinical Psychology). Note that psychoanalytic therapy also was listed as “controversial.”

c Psychotherapies for alcohol use disorders are those with support in a majority of reviews, as identified via the systematic analysis of Miller and colleagues (2005). Twelve-step facilitation was added based on published empirical support (e.g., Project MATCH Research Group 1997, 1998; McKellar et al. 2003; Tonigan 2009).

**Table 3:** Comparative Balance of Comorbidity Treatment Models

**Model**	**Description**	**Advantages**	**Disadvantages**
**Sequential**	Treatment of one disorder followed by treatment of the second comorbid disorder	Can accommodate differential treatment interests among anxiety versus alcohol treatment seekers Allows for hypothesis testing of causal relationships among presenting symptoms If treatment of first disorder (e.g. alcohol use disorders (AUD) leads to reduction in symptoms of second disorder (e.g. anxiety reduction), unnecessary treatment of second disorder may be avoided	Case coordination can be complicated if different providers or treatment settings are involved Mutual maintenance pattern may compromise treatment gains for first disorder treated, leading to greater risk for relapse Implicit communication to clients that one disorder is more important than the other
**Parallel/simultaneous**	Specific treatment of both comorbid disorders at the same time but not necessarily by the same provider or in the same treatment facility	Roughly equivalent attention given to both disorders Both disorders are treated by experts in their respective areas Recognition that each comorbid disorder needs treatment attention, to reduce risk for relapse to each disorder being treated based on mutual maintenance pattern	Case coordination can be complicated if different providers or treatment settings are involved Clients may become overwhelmed by excessive demands of simultaneous treatment of two (or more) disorders Can ignore functional interrelationship among comorbid disorders
**Integrated**	Both disorders are treated, or at least monitored simultaneously, by a single qualified provider	Treatment addresses the functional interrelationship of comorbid disorders Both disorders are treated by the same provider at the same time, which eliminates case coordination difficulties associated with other treatment models Treatment efficiency is potentially maximized	Lack of professionals qualified to treat both disorders, especially considering the wide range of potential unique anxiety–AUD combinations Clients seeking treatment for one problem may have no interest in addressing the other comorbid disorder, which can compromise therapeutic alliance Assumption of functional interrelationship between comorbid disorders may not fit all cases

## Footnotes

1 For these analyses, anxiolytic use was standardized by converting all reported benzodiazepine use into chlordiazepoxide equivalents.
